# The changes of Treg and Th17 cells relate to serum 25(OH)D in patients with initial-onset childhood systemic lupus erythematosus

**DOI:** 10.3389/fped.2023.1228112

**Published:** 2023-08-23

**Authors:** Li-jun Jiang, Zan-hua Rong, Hui-feng Zhang

**Affiliations:** Department of Pediatrics, The Second Hospital of Hebei Medical University, Shijiazhuang, China

**Keywords:** 25(OH)D, SLE, Treg, Th17, cytokines

## Abstract

**Background:**

T helper 17 (Th17) cells and regulatory T cells (Treg) are known to play a crucial role in the pathogenesis of systemic lupus erythematosus (SLE). Improving the balance between Treg and Th17 cells can be a promising new therapeutic target in SLE patients. Vitamin D has a significant impact on the immune inflammatory process and the immune cells involved in this process. The purpose of this study is to investigate the relationship between Th17, Treg, cytokines, and serum 25 hydroxyvitamin D [25(OH)D] in patients with initial-onset childhood SLE.

**Methods:**

A total of 82 children aged <18 years with initial-onset SLE were included, as well as 60 healthy subjects during the same period at the Pediatrics Department of the Second Hospital of Hebei Medical University. The chemiluminescence method was performed to detect serum 25(OH)D levels. Flow cytometry was used to evaluate Treg and Th17 cells. An enzyme-linked immunosorbent assay kit was used to evaluate plasma interleukin (IL)-23, IL-17, IL-10, IL-6, and tumor necrosis factor alpha (TNF-α) concentrations.

**Result:**

The serum 25(OH)D levels in patients with initial-onset childhood SLE were significantly lower than those in the healthy controls. The proportion of lupus nephritis (LN) was higher in the vitamin D insufficiency group (71.4%) compared with the vitamin D sufficiency group (30.3%) (*p *< 0.05). The SLE disease activity index (SLEDAI) was higher in the vitamin D insufficiency group (median = 14) than that in the vitamin D sufficiency group (median = 9) (*p *< 0.05).The 25(OH)D level was positively correlated with the Treg ratio (*r* = 0.337, *p *= 0.002), and it was negatively correlated with the Th17 cell ratio (*r* = −0.370, *p *= 0.001). The serum 25(OH)D level had a negative correlation with IL-23 (*r* = −0.589, *p *< 0.001), IL-17(*r* = −0.351, *p *= 0.001), TNF-α (*r* = −0.283, *p *= 0.01), IL-6 (*r* = −0.392, *p *< 0.001), and IL-10 (*r* = −0.313, *p *= 0.004) levels.

**Conclusion:**

The serum 25(OH)D levels decreased in patients with initial-onset childhood SLE. There was a negative correlation between the serum 25(OH)D levels and SLEDAI. The serum 25(OH)D levels in patients with initial-onset childhood SLE were negatively correlated with the Th17 ratio and related cytokines, while positively correlated with the Treg ratio.

## 1. Introduction

Systemic lupus erythematosus (SLE) is a chronic systemic autoimmune disease that causes chronic inflammation and damages multiple tissues and organs, including the central nervous system, skin mucosa, cardiovascular system, kidneys, and joints. The pathogenesis of SLE is not fully understood. SLE is characterized by polyclonal activation of T and B lymphocytes. In addition to an imbalance of T helper 1 (Th1) and T helper 2 (Th2) cells, the regulatory T cells (Treg) and T helper 17 (Th17) cells are known to play a crucial role in the pathogenesis of SLE (1). Studies have found that quantity anomalies or/and functional defects of Treg and Th17 cells were associated with flares and organ damages in SLE patients (2). Th17 cells secrete a profile of potent pro-inflammatory cytokines, including interleukin-17 (IL-17), and potent tumor necrosis factor alpha (TNF-α) and interleukin-6 (IL-6) upon certain stimulation (3). Interleukin-23 (IL-23) is an important cytokine that promotes the secretion of interleukin-17 by Th17 cells to maintain pathological status by combining with IL-23 receptor (4). Although it is widely believed that Treg cells play a preventive role in autoimmunity, the data on SLE are inconsistent (5). Interleukin-10 (IL-10) is secreted not only by Th2 cells but also by Treg cells. The differentiation and proliferation of Treg and Th17 cells are regulated by multiple cytokines including IL-10, IL-23, IL-17, and IL-6 (6). Regulating the balance between Treg and Th17 cells will be a promising new therapeutic target in SLE patients.

Vitamin D is an important steroid hormone that has significant effects on bone health and the cardiovascular system (7). Vitamin D also has some non-classical effects, such as immune modulatory effects (8). Many studies have found that most patients with autoimmune diseases worldwide suffer from vitamin D deficiency. These studies have also emphasized the relationship between decreased serum vitamin D levels and disease activity in SLE and rheumatoid arthritis (9–11). Vitamin D has great impact on immune cells as well as the inflammatory cascade. The receptors of Vitamin D are commonly accessible for many adaptive immune cells including T cells, B cells, macrophages, and dendritic cells (12).

Whether vitamin D can act on Treg and Th17 cells remains largely unexplored. Therefore, the purpose of this study is to investigate the relationship between Th17, Treg, cytokines, and serum 25 hydroxyvitamin D [25(OH)D] in patients with initial-onset childhood SLE.

## 2. Patients and methods

### 2.1. Study subjects

A total of 82 children aged <18 years with initial-onset SLE who were admitted to the Pediatrics Department of the Second Hospital of Hebei Medical University between April 2020 and February 2023 were included in this study. All patients met the 1997 American College of Rheumatology (ACR) classification criteria for SLE (13) or the 2012 Systemic Lupus Erythematosus International Collaborating Clinics (SLICC) classification criteria for SLE (14). The disease activity was assessed using the SLE Disease Activity Index-2000 (SLEDAI-2K). The exclusion criteria were as follows: certain diseases that affect vitamin D metabolism (gastrointestinal surgery, liver metabolic diseases, tumors, etc.); and vitamin D supplementation by oral medication within the past 3 months. This study involved 60 healthy subjects during the same period as healthy controls (HC). This study was approved by the ethics committee of the Second Hospital of Hebei Medical University (protocol number 2021-R307).

### 2.2. Laboratory examinations

Laboratory examinations included routine blood tests, 24-h urine protein, erythrocyte sedimentation rate (ESR), liver function, renal function, complement 3 (C3), complement 4 (C4), antinuclear antibody, double-stranded deoxyribonucleic acid (dsDNA), serum calcium, and serum phosphorus.

### 2.3. Determination of serum 25(OH)D level

Blood was collected between 6:00 and 7:00 in the morning, and the children were fasted from food and water overnight before the blood samples were collected. The chemiluminescence method was performed for the detection of serum 25(OH)D levels, the kit was provided by Siemens Healthcare Diagnostics Inc. (USA), and the analysis was done using an ADVIA Centaur XP automatic chemiluminescence immunoassay analyzer. A vitamin D insufficiency was defined as serum 25(OH)D level of  < 20 ng/ml, and a vitamin D sufficiency was defined as serum 25(OH)D level of **_ _**≥ 20 ng/ml.

### 2.4. Flow cytometry

#### 2.4.1. Sample and cell preparations

All participants fasted from water after 12 p.m. the previous day, and peripheral venous blood samples of approximately 5 ml were collected between 6:00 and 7:00 in the morning. Blood samples were anticoagulated with ethylenediaminetetraacetic acid dipotassium (EDTA-K2), which was used to isolate and identify Treg and Th17 cell subsets. Peripheral blood mononuclear cells (PBMCs) were obtained through Ficoll density gradient. PBMCs were suspended at a density of 2 × 10^6^ cells/ml on a complete culture medium (RPMI 1640 supplemented with 100 µg/ml streptomycin, 100 U/ml penicillin, 2 mM glutamine, and 10% heat-inactivated fetal calf serum) to obtain and analyze Th17 cell subset. The cell suspension was transferred to 24-well culture plates with a concentration of 25 ng/ml of phorbol ethyl ester (PMA), 1.7 ml of moneomycin (MN), and 1 ml of ionomycin (LC), and then incubated at 37°C under a 5% CO_2_ environment for 4 h. For Treg cells analysis, PBMCs were suspended at a density of 2 × 10^7^ cells/ml.

#### 2.4.2. Surface and intracellular staining

To analyze Th17 cell subset, the cells were fixed and permeabilized according to the manufacturer's instructions, and then intracellularly stained with PE-conjugated anti-IL-17 monoclonal antibodies. Th17 cells were labeled as CD4 + IL-17A+. For Treg analysis, the cells were surface-stained, and then fixed permeabilized, then stained with PE anti-human Foxp3 according to the manufacturer's instructions. Treg cells were labeled as CD4 + CD25 + FoxP3+. Homotypic controls were used to verify specificity and perform compensation correction. All antibodies were provided by eBioscience. Stained cells were analyzed by flow cytometry analysis using a FACSCalibur flow cytometer (BD biosciences) with FlowJo software (Tree Star, San Carlos, CA, USA).

#### 2.4.3. Enzyme-linked immunosorbent assay

A total of 3 ml of blood was collected and anticoagulated with EDTA-K2 to evaluate cytokines. Plasma IL-23, IL-17, TNF-α, IL-6, and IL-10 concentrations were determined by using human IL-23, IL-17, TNF-α, IL-6, and IL-10 enzyme-linked immunosorbent assay (ELISA) kit (Elabscience, Elabscience Biotechnology Co., Ltd.).

### 2.5. Statistical analysis

The data were statistically analyzed by using the SPSS version23.0 program. The data were presented as the mean ± standard deviation (SD) or median, and the categorical variables were expressed as frequencies and percentages. The rates were compared between two or more groups using chi-square test or Fisher's exact test. A non-parametric Mann–Whitney *U* test was used to compare the data between groups. Pearson correlation analysis was used for variables that conformed to a normal distribution, and Spearman correlation analysis was used for variables that did not conform to a normal distribution. Statistically significant was defined as *p*-value less than 0.05.

## 3. Result

### 3.1. Serum 25(OH)D level

**Figure 1:**
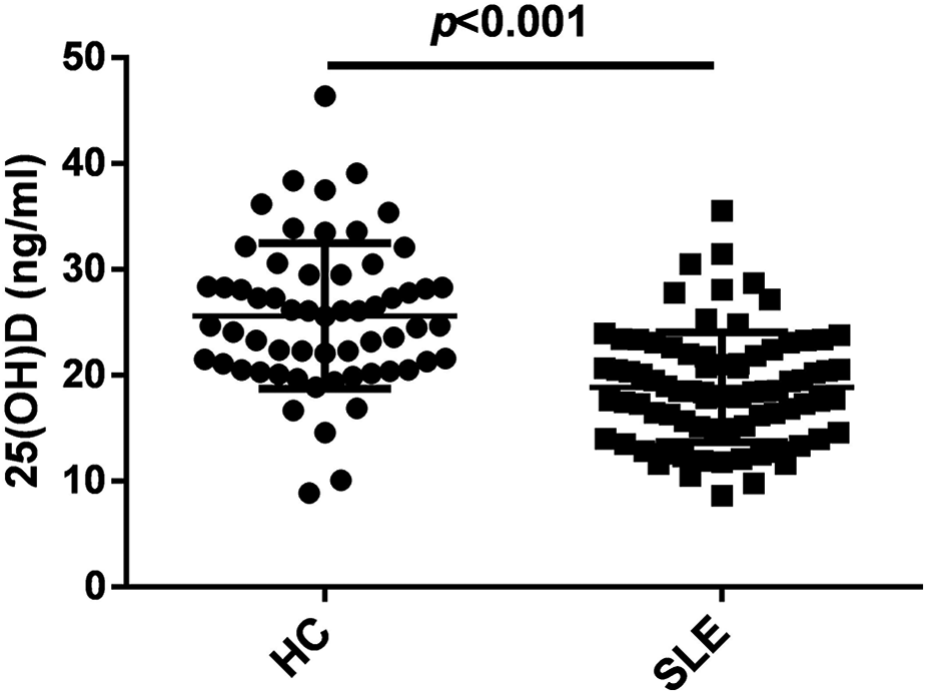
Serum 25 hydroxyvitamin D [25(OH)D] levels were decreased in patients with initial-onset childhood SLE, *n* = 60 for HC and *n* = 82 for SLE.

As shown in Figure 1, the serum 25(OH)D levels in patients with initial-onset childhood SLE were significantly lower than those in the healthy control group (25.60 ± 6.87 ng/ml for HC, 18.86 ± 5.18 ng/ml for SLE).

### 3.2. Comparison of clinical and laboratory findings in pediatric SLE patients with different 25(OH)D levels

In this study, the patients with initial-onset childhood SLE were divided into two groups based on their serum 25(OH)D levels. The clinical manifestations and laboratory parameters were compared between the two groups. The proportion of lupus nephritis (LN) was higher in the vitamin D insufficiency group (71.4%) compared with the vitamin D sufficiency group (30.3%) (*p *< 0.05). SLEDAI was higher in the vitamin D insufficiency group (median = 14) than that in the vitamin D sufficiency group (median = 9) (*p *< 0.05). The probability of pulmonary involvement and anemia was higher in the vitamin D insufficiency group. The SLEDAI-2K score was higher in the vitamin D insufficiency group (median = 14) than that in the vitamin D sufficiency group (median = 9) (*p *< 0.05), indicating higher disease activity in SLE. Compared with the vitamin D sufficiency group (0.54 ± .024 g/L), SLE patients in the vitamin D insufficiency group had lower levels of C3 (0.39 ± 0.27 g/L) (*p *< 0.05). It was worth noting that SLE children with insufficient vitamin D had lower serum calcium levels (Table 1).

**Table 1:** Demographic, clinical, and laboratory characteristics of the studied groups.

Characteristic	Total	25(OH)D < 20 ng/ml	25(OH)D ≥ 20 ng/ml	*p*
Female sex, n/N (%)	67/82 (81.7%)	40/49 (81.6%)	27/33 (81.8%)	0.983
Age (years), mean ± SD	11.4 ± 2.2	11.6 ± 2.4	11.2 ± 2.0	0.320
Lupus nephritis, *n* (%)	45 (54.9%)	35 (71.4%)	10 (30.3%)	**<0**.**001**
Arthritis, *n* (%)	22 (26.8%)	14 (28.6%)	8 (24.2%)	0.664
Mucocutaneous, *n* (%)	39 (47.6%)	23 (46.9%)	16 (48.5%)	0.891
Vasculitis, *n* (%)	1 (1.2%)	1 (2%)	0 (0%)	0.598
Serositis, *n* (%)	8 (9.8%)	7 (14.3%)	1 (3%)	0.092
Neurologic, *n* (%)	16 (19.5%)	9 (18.4%)	7 (21.2%)	0.750
Pulmonary, *n* (%)	13 (15.9%)	11 (22.4%)	2 (6.1%)	**0**.**046**
SLEDAI, median (range)	12 (2–33)	14 (2–33)	9 (3–31)	**0**.**048**
Leukopenia (<4 × 10^9^/L), *n* (%)	42 (51.2%)	21 (42.9%)	21 (63.6%)	0.065
Anemia (<110 g/L), *n* (%)	46 (56.1%)	33 (67.3%)	13 (39.4%)	**0**.**012**
Thrombocytopenia (<100 × 10^9^/L), *n* (%)	27 (32.9%)	20 (40.8%)	7 (21.2%)	0.064
Proteinuria, *n* (%)	43 (52.4%)	35 (71.4%)	8 (24.2%)	<0.001
C3 (g/L), mean ± SD	0.45 ± 0.27	0.39 ± 0.27	0.54 ± 0.24	**0**.**004**
C4 (g/L), mean ± SD	0.07 ± 0.06	0.07 ± 0.06	0.08 ± 0.05	0.055
Positive anti-dsDNA, *n* (%)	52 (63.4%)	33 (67.3%)	19 (57.6%)	0.368
Serum calcium (mmol/L), mean ± SD	2.17 ± 0.16	2.11 ± 0.16	2.26 ± 0.12	**<0**.**001**
Serum phosphorus (mmol/L), mean ± SD	1.55 ± 0.43	1.61 ± 0.47	1.46 ± 0.33	0.356

Statistically significant results are highlighted in bold.

### 3.3. Correlations of 25(OH)D levels with the clinical and laboratory parameters

The 25(OH)D levels were positively correlated with C3 (*r* = 0.303, *p *= 0.006) and C4 (*r* = 0.225, *p *= 0.042), while the 25(OH)D levels were negatively correlated with 24-h urinary protein (*r* = −0.423, *p *< 0.001) (Table 2).

**Table 2:** Correlations of 25(OH)D levels with the clinical and laboratory parameters.

	*R* (Spearman correlation)	*p*
24-h urinary protein	−0.423	**<0**.**001**
C3	0.303	**0**.**006**
C4	0.225	**0**.**042**
SLEDAI	−0.168	0.131

Statistically significant results are highlighted in bold.

### 3.4. Correlations of the Treg ratio with the clinical and laboratory parameters

The Treg ratio was positively correlated with C4 (*r* = 0.281, *p *= 0.011), while it was negatively correlated with 24-h urinary protein (*r* = −0.261, *p *= 0.018) and SLEDAI (*r* = −0.268, *p *= 0.015) (Table 3).

**Table 3:** Correlations of the Treg ratio with the clinical and laboratory parameters.

	*R* (Spearman correlation)	*p*
24-h urinary protein	−0.261	**0**.**018**
C3	0.212	0.056
C4	0.281	**0**.**011**
SLEDAI	−0.268	**0**.**015**

Statistically significant results are highlighted in bold.

### 3.5. Correlations of the Th17 cell ratio with the clinical and laboratory parameters

The Th17 cell ratio was positively correlated with 24-h urinary protein (*r* = 0.277, *p *= 0.012) and SLEDAI (*r* = 0.287, *p *= 0.009), while it was negatively correlated with C3 (*r* = −0.257, *p *= 0.02) (Table 4).

**Table 4:** Correlations of the Th17 cell ratio with the clinical and laboratory parameters.

	*R* (Spearman correlation)	*p*
24-h urinary protein	0.277	**0**.**012**
C3	−0.257	**0**.**02**
C4	−0.155	0.164
SLEDAI	0.287	**0**.**009**

Statistically significant results are highlighted in bold.

### 3.6. The relationship between 25(OH)D levels and the proportion of Treg and Th17 cells

**Figure 2:**
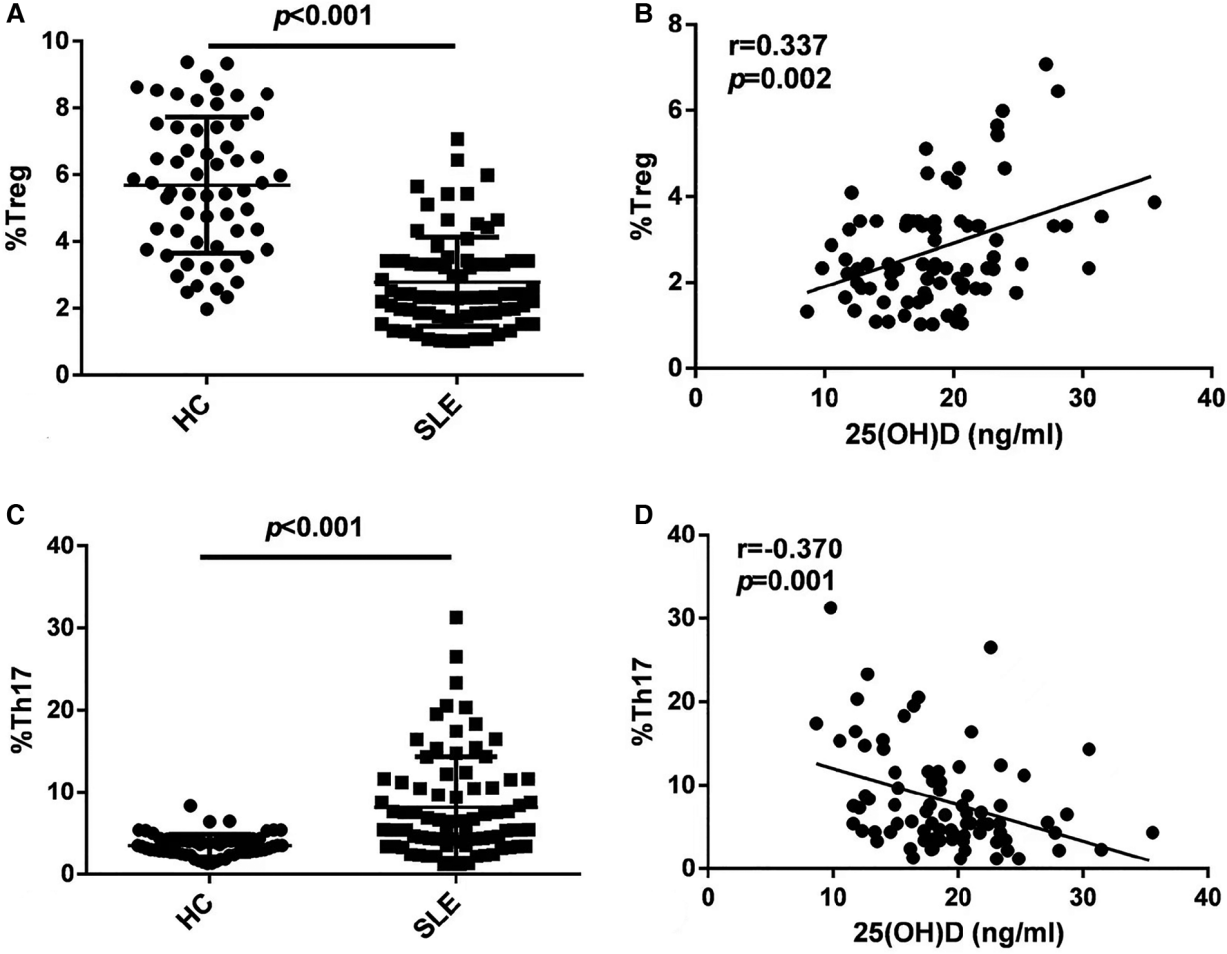
Flow cytometry analysis of Treg subset (A) and Th17 cell subset (C) in peripheral blood, *n* = 60 for HC and *n* = 82 for SLE. The correlation analysis of 25(OH)D levels and Treg subset (B) and Th17 cell subset (D) in peripheral blood in patients with initial-onset childhood SLE (*n* = 82).

As shown in Figure 2A, the Treg ratio in children with initial-onset childhood SLE decreased (5.69 ± 2.03 for HC, 2.79 ± 1.33 for SLE). The 25(OH)D levels were positively correlated with the Treg ratio (*r* = 0.337, *p *= 0.002) (Figure 2B). As shown in Figure 2C, the Th17 cell ratio in patients with initial-onset childhood SLE increased (3.52 ± 1.36 for HC, 8.16 ± 6.16 for SLE). The 25(OH)D levels were negatively correlated with the Th17 cell ratio (*r* = −0.370, *p *= 0.001) (Figure 2D).

### 3.7. Negative correlation between serum 25(OH)D and serum levels of cytokines

**Figure 3:**
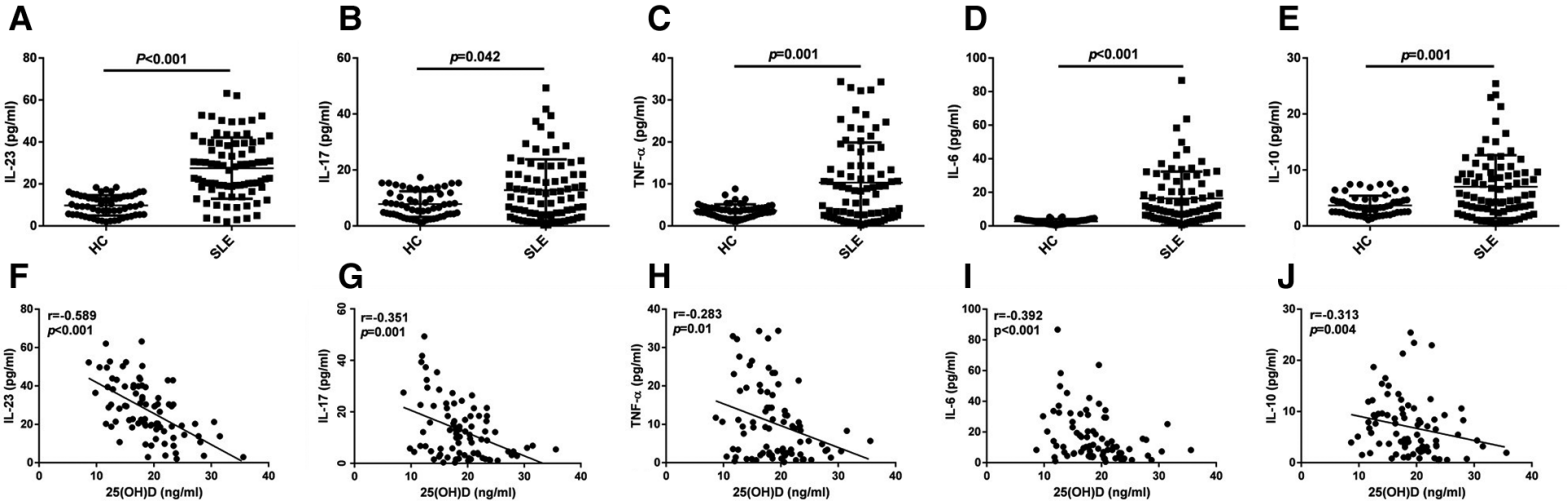
ELISA analysis of IL-23, IL-17, TNF-α, IL-6, and IL-10 (**A–E**), *n* = 60 for HC and *n* = 82 for SLE. The correlation analysis of serum 25(OH)D level and IL-23, IL-17, TNF-α, IL-6, and IL-10 in serum from patients with initial-onset childhood SLE (**F–J**) (*n* = 82).

The ELISA results showed a significant increase of the levels of IL-23, IL-17, TNF-α, IL-10, and IL-6 in patients with initial-onset childhood SLE (Figures 3A–E, 9.78 ± 4.84 pg/ml vs. 27.53 ± 14.55 pg/ml; 7.80 ± 4.59 pg/ml vs. 12.77 ± 11.00 pg/ml; 3.62 ± 1.55 pg/ml vs.10.32 ± 9.57 pg/ml; 3.66 ± 1.73 pg/ml vs.6.99 ± 5.63 pg/ml; 2.72 ± 1.33 pg/ml vs.18.86 ± 15.98 pg/ml). The serum 25(OH)D levels had a negative correlation with IL-23 (*r* = −0.589, *p *< 0.001), IL-17(*r* = −0.351, *p *= 0.001), TNF-α (*r* = −0.283, *p *= 0.01), IL-6 (*r* = −0.392, *p *< 0.001), and IL-10 (*r* = −0.313, *p *= 0.004) levels (Figures 3F–J).

## 4. Discussion

SLE is a chronic autoimmune disease distinguished by auto-antibodies development and persistent inflammation that damages multiple organs. The clinical manifestations and severity of pediatric SLE are not completely the same as those of adult SLE, with childhood SLE having more severe clinical manifestations and being more prone to involving important organs compared with adult SLE. However, research on childhood SLE has not been widely reported (12).

The increase of Th17 and the decrease of Treg subsets were reported to be the main factors related to organ damages and auto-antibodies production in SLE patients (15). The elevation of the proportion of Th17 cells with pro-inflammatory effects was reported to be positively related to the disease activity of SLE (15). Treg cells have immunosuppressive function and can induce and maintain the self-immune tolerance of the body. The decrease of Treg and its dysfunction play a very important role in the pathogenesis of SLE (1). Injecting Treg into SLE mice could alleviate inflammation and reduce tissue damage (1). Our study found that the ratio of Th17 cells significantly elevated in initial-onset childhood SLE, while the proportion of Treg significantly decreased compared with healthy controls. However, there have been reports of an increase of the percentage of Treg and Th17 cells rather than a decrease of the number of Treg in SLE patients (2). The research results on the ratio of Treg in SLE patients are inconsistent. Therefore, it is currently believed that not only abnormal proportions but, more importantly, abnormal functions of Treg are involved in the pathogenesis of SLE. Reports confirmed that the mTOR signaling pathway regulates the proliferation, differentiation, and functions of Treg cells (16). Specifically, mTORC1 promotes the expansion of pro-inflammatory lymphocyte subsets such as Th17; mTORC2 drives the proliferation of T follicle helper cells, promoting the activation of B cells and generation of auto-antibodies (17). Both mTORC1 and mTORC2 can control the differentiation and maturation of CD4 + CD25 + Foxp3 + Treg cells (17). In SLE patients, the abnormal metabolism of T cells, including high mTOR activation, increased glutaminolysis, active lipid synthesis, and enhanced glycolysis, all contribute to the differentiation and function of Th17. The metabolic disorder of T cell is a potential mechanism for Th17/Treg imbalance in SLE patients (1).

The serum 25(OH)D levels for children with SLE were obviously lower than those for healthy control children. Consistent with our findings, multiple studies worldwide have found lower levels of 25(OH)D in adults with SLE (10). Although insufficiency and/or deficiency of vitamin D have been reported in children with SLE (11, 12), there is relatively few studies on vitamin D levels in pediatric SLE. There are various reasons for the decrease of serum vitamin D levels in patients with SLE. Vitamin D is mainly synthesized through the epidermal layer of the skin after ultraviolet exposure (7). The main measures for SLE patients to avoid photosensitivity are sunshade and using sunscreen, but these are also risk factors for vitamin D deficiency (10). Cusack et al. (18) found that using sunscreen had an impact on the levels of 25(OH)D depending on using time. Drugs used to treat SLE may exacerbate vitamin D deficiency, such as glucocorticoids reducing intestinal absorption of vitamin D and accelerating the catabolism of 25(OH)D and l,25(OH)_2_D by enhancing 24-hydroxylase activity (19, 20). It is reported that proteinuria had a great impact on the concentration of vitamin D, which may be due to the loss of vitamin D-binding protein (DBP) caused by kidney damage in SLE (21). Our study found that the levels of serum 25(OH)D were negatively correlated with the quantification of urinary protein. Young et al. (22) found that vitamin D deficiency was driven by genetic factors, not just due to sun shielding. CYP24A1 rs4809959 modified the association of 25(OH)D and SLE. Vitamin D receptor (VDR) polymorphisms are associated with higher risk of SLE among different races, especially among Asians and Africans (23). Clinical studies found that supplementing vitamin D has an improvement effect on reducing disease activity and alleviating fatigue in patients with SLE (24).

Children with initial-onset SLE had elevated ratios of Thl7 cells and decreased ratios of Treg in their peripheral blood. The levels of 25(OH)D in patients with initial-onset childhood SLE were negatively correlated with the proportion of Th17 cells and positively correlated with the proportion of Treg cells. Th17 cell is a new CD4 + T helper cell subset discovered in recent years. Its proliferation and differentiation are different from Th1 and Th2 cells. Th17 expresses specific nuclear transcription factor rROR γT and can secrete specific cytokines such as IL-17 and IL-22 (25). Vitamin D3 signaling inhibits Th17 cell differentiation. Vitamin D3 acts on Th17 cells, inhibiting the expression of IL-22, IL-17, chemokine receptor CCR6, TNF-α, and IFN-γ, thereby preventing Th17 cells from migrating to inflammatory tissues (26, 27). The 1,25(OH)_2_D binds the vitamin D receptor to vitamin D response element (VDRE) in the *FoxP3* gene and then directly upregulates the expression of Treg marker *FoxP3* (24). There is evidence to suggest that 1,25(OH)_2_D can upregulate the expression of *FoxP3* in immature CD4 + T cells and induce differentiation of Treg cells, leading to an increase in the functional expression of regulatory markers such as IL-10 and cytotoxic T lymphocyte-associated protein 4 (CTLA-4) (28). Research studies have also shown that Th2 and Th17 cells are transformed into plastic phenotypes through the action of 1,25(OH)_2_D (27). The 1,25(OH)_2_D induces the phenotype of Treg by upregulating the expression of *FoxP3* and *CLTA4* genes, while downregulating the expression of *IL17A* genes (29).

The levels of cytokines related to Th17 cells, such as IL-6, TNF-α, IL-23, and IL-17, were significantly elevated in patients with initial-onset childhood SLE. The levels of cytokines associated to Treg cells, such as IL-10, were also elevated. The levels of IL-6, TNF-α, IL-23, IL-17, and IL-10 were negatively correlated with the serum 25(OH)D level. Research studies show that after vitamin D treatment, Treg and Th2 cells increased, and Th17 and Th1 cells decreased inconsistently (30, 31). IL-6 inhibits the expression of *Foxp3* during Treg differentiation (32). Relative studies showed that the levels of inflammatory cytokines, such as IL-6, IL-1, IL-18, and TNF-α, were significantly reduced with the vitamin D treatment group in SLE patients. On the other hand, vitamin D treatment upregulated IL-10 expression (33, 34). A study found a positive correlation between elevated serum 25(OH)D and elevated IL-10. The author showed that after 8 weeks of vitamin D treatment, the levels of IL-10 significantly increased, while there was no significant change in the level of TGF-β1 in multiple sclerosis patients (35). However, our study showed that the levels of IL-10 increased in patients with initial-onset childhood SLE, and a negative correlation can be observed between serum vitamin D levels and blood IL-10 levels, which was inconsistent with other studies.

Vitamin D, as an immune regulatory factor, participates in innate and adaptive immunity (36). The immune regulatory role of vitamin D in autoimmune diseases has always been a focus of research (37). Multiple epidemiological studies worldwide have found vitamin D deficiency or insufficiency in various autoimmune diseases (37). Vitamin D not only regulates Th17 and Treg cell differentiation, but also acts on other T lymphocyte subsets, B cells, dendritic cells, etc. Both T cells and B cells express VDR, which is an important target for vitamin D to exert immune regulation. Vitamin D induces tolerance phenotype by acting on antigen-presenting cell, monocyte, natural killer cell, and dendritic cells, enhance chemotaxis of neutrophil (38).

Osteoporosis can occur in patients with SLE, including juvenile patients, possibly due to chronic inflammation affecting bone metabolism and the use of glucocorticoids and other drugs (39). It is recommended to monitor the calcium and phosphorus metabolism as well as vitamin D levels in pediatric SLE patients.

Our research has limitations. The sample size included in this study is not large enough and cannot be subjected to a stratified analysis. We only studied Treg and Th17 cells and related cytokines, but did not include other lymphocyte subpopulations. We studied the relation between the serum 25(OH)D levels and the ratio of Treg and Th17 cells in peripheral blood, but did not conduct a double-blind controlled randomized study to observe the changes in Treg and Th17 cells after vitamin D treatment. The molecular mechanism by which vitamin D acts on Treg and Th17 cells in SLE patients is not yet well understood. These will be explored in our future research.

## 5. Conclusion

The imbalance of Treg and Th17 cell differentiation leads to the suppression of immune function and promotes the development of SLE. In patients with initial-onset childhood SLE, the changes of serum vitamin D levels can affect the proportion of Treg cell subset and TH17 cell subset and can also affect the levels of cytokines related to these T cell subpopulations. The molecular mechanism of action of vitamin D and lymphocyte subpopulations in SLE is complex. Further exploration should be conducted on the role and mechanism of vitamin D in regulating Th17 and Treg subsets, providing a basis for immunotherapy in pediatric SLE.

## Data Availability

The original contributions presented in the study are included in the article/supplementary material, further inquiries can be directed to the corresponding author.

## References

[1] Shan J Jin H Xu Y. T cell metabolism: a new perspective on Th17/Treg cell imbalance in systemic lupus erythematosus. Front Immunol. (2020) 11:1027. 10.3389/fimmu.2020.01027 32528480 PMC7257669

[2] Handono K Firdausi SN Pratama MZ Endharti AT Kalim H. Vitamin A improve Th17 and Treg regulation in systemic lupus erythematosus. Clin Rheumatol. (2016) 35(3):631–8. 10.1007/s10067-016-3197-x 26852315

[3] Talaat RM Mohamed SF Bassyouni IH Raouf AA. Th1/Th2/Th17/Treg cytokine imbalance in systemic lupus erythematosus (SLE) patients: correlation with disease activity. Cytokine. (2015) 72(2):146–53. 10.1016/j.cyto.2014.12.027 25647269

[4] Philippot Q Ogishi M Bohlen J Puchan J Arias AA Nguyen T Human IL-23 is essential for IFN-γ-dependent immunity to mycobacteria. Sci Immunol. (2023) 8(80):eabq5204. 10.1126/sciimmunol.abq5204 36763636 PMC10069949

[5] Li Y Tang D Yin L Dai Y. New insights for regulatory T cell in lupus nephritis. Autoimmun Rev. (2022) 21(8):103134. 10.1016/j.autrev.2022.103134 35690245

[6] Chen B Jin L. Low serum level of 25-OH vitamin D relates to Th17 and Treg changes in colorectal cancer patients. Immun Inflamm Dis. (2022) 10(11):e723. 10.1002/iid3.723 36301026 PMC9597490

[7] Shoenfeld Y Giacomelli R Azrielant S Berardicurti O Reynolds JA Bruce IN. Vitamin D and systemic lupus erythematosus—the hype and the hope. Autoimmun Rev. (2018) 17(1):19–23. 10.1016/j.autrev.2017.11.004 29108830

[8] Salman-Monte TC Torrente-Segarra V Vega-Vidal AL Corzo P Castro-Dominguez F Ojeda F Bone mineral density and vitamin D status in systemic lupus erythematosus (SLE): a systematic review. Autoimmun Rev. (2017) 16(11):1155–9. 10.1016/j.autrev.2017.09.011 28899800

[9] Correa-Rodríguez M Pocovi-Gerardino G Callejas-Rubio JL Ríos-Fernández R Martín-Amada M Cruz-Caparrós MG Vitamin D levels are associated with disease activity and damage accrual in systemic lupus erythematosus patients. Biol Res Nurs. (2021) 23(3):455–63. 10.1177/1099800420983596 33380211

[10] Jiang Z Pu R Li N Chen C Li J Dai W High prevalence of vitamin D deficiency in Asia: a systematic review and meta-analysis. Crit Rev Food Sci. (2023) 63(19):3602–11. 10.1080/10408398.2021.1990850 34783278

[11] Cheng KH Tsai MC Fu LS. The correlation between VitD3 levels and the disease activity of childhood-onset systemic lupus erythematosus. J Chin Med Assoc. (2022) 85(5):627–32. 10.1097/JCMA.0000000000000702 35506950 PMC12755709

[12] Abo-Shanab AM Kholoussi S Kandil R Dorgham D. Cytokines, 25-OH Vit D and disease activity in patients with juvenile-onset systemic lupus erythematosus. Lupus. (2021) 30(3):459–64. 10.1177/0961203320973068 33183127

[13] Hochberg MC. Updating the American College of Rheumatology revised criteria for the classification of systemic lupus erythematosus. Arthritis Rheum. (1997) 40(9):1725. 10.1002/art.1780400928 9324032

[14] Petri M Orbai AM Alarcón GS Gordon C Merrill JT Fortin PR Derivation and validation of the systemic lupus international collaborating clinics classification criteria for systemic lupus erythematosus. Arthritis Rheum. (2012) 64(8):2677–86. 10.1002/art.34473 22553077 PMC3409311

[15] Wang D Lei L. Interleukin-35 regulates the balance of Th17 and Treg responses during the pathogenesis of connective tissue diseases. Int J Rheum Dis. (2021) 24(1):21–7. 10.1111/1756-185X.13962 32918357

[16] Zhao X Wang S Wang S Xie J Cui D. mTOR signaling: a pivotal player in Treg cell dysfunction in systemic lupus erythematosus. Clin Immunol. (2022) 245:109153. 10.1016/j.clim.2022.109153 36265758

[17] Yang J Yang X Zou H Li M. Oxidative stress and Treg and Th17 dysfunction in systemic lupus erythematosus. Oxid Med Cell Longev. (2016) 2016:2526174. 10.1155/2016/2526174 27597882 PMC4997077

[18] Cusack C Danby C Fallon JC Ho WL Murray B Brady J Photoprotective behaviour and sunscreen use: impact on vitamin D levels in cutaneous lupus erythematosus. Photodermatol Photo. (2008) 24(5):260–7. 10.1111/j.1600-0781.2008.00373.x 18811868

[19] Kamen DL. Vitamin D in lupus—new kid on the block? Bull NYU Hosp Jt Dis. (2010) 68(3):218–22. 20969555 PMC4185297

[20] Dhawan P Christakos S. Novel regulation of 25-hydroxyvitamin D3 24-hydroxylase (24(OH)ase) transcription by glucocorticoids: cooperative effects of the glucocorticoid receptor, C/EBP beta, and the vitamin D receptor in 24(OH)ase transcription. J Cell Biochem. (2010) 110(6):1314–23. 10.1002/jcb.22645 20564225

[21] Sahebari M Nabavi N Salehi M. Correlation between serum 25(OH)D values and lupus disease activity: an original article and a systematic review with meta-analysis focusing on serum VitD confounders. Lupus. (2014) 23(11):1164–77. 10.1177/0961203314540966 24961748

[22] Young KA Munroe ME Guthridge JM Kamen DL Niewold TB Gilkeson GS Combined role of vitamin D status and CYP24A1 in the transition to systemic lupus erythematosus. Ann Rheum Dis. (2017) 76(1):153–8. 10.1136/annrheumdis-2016-209157 27283331 PMC5360632

[23] Ritterhouse LL Crowe SR Niewold TB Kamen DL Macwana SR Roberts VC Vitamin D deficiency is associated with an increased autoimmune response in healthy individuals and in patients with systemic lupus erythematosus. Ann Rheum Dis. (2011) 70(9):1569–74. 10.1136/ard.2010.148494 21586442 PMC3149865

[24] Bishop EL Ismailova A Dimeloe S Hewison M White JH. Vitamin D and immune regulation: antibacterial, antiviral, anti-inflammatory. JBMR Plus. (2021) 5(1):e10405. 10.1002/jbm4.10405 32904944 PMC7461279

[25] Kono M. New insights into the metabolism of Th17 cells. Immunol Med. (2023) 46(1):15–24. 10.1080/25785826.2022.2140503 36326754

[26] Dankers W Davelaar N van Hamburg JP van de Peppel J Colin EM Lubberts E. Human memory Th17 cell populations change into anti-inflammatory cells with regulatory capacity upon exposure to active vitamin D. Front Immunol. (2019) 10:1504. 10.3389/fimmu.2019.01504 31379807 PMC6651215

[27] Hafkamp FMJ Taanman-Kueter EWM van Capel TMM Kormelink TG de Jong EC. Vitamin D3 priming of dendritic cells shifts human neutrophil-dependent Th17 cell development to regulatory T cells. Front Immunol. (2022) 13:872665. 10.3389/fimmu.2022.872665 35874744 PMC9301463

[28] Chambers ES Suwannasaen D Mann EH Urry Z Richards DF Lertmemongkolchai G 1α,25-dihydroxyvitamin D3 in combination with transforming growth factor-β increases the frequency of Foxp3+ regulatory T cells through preferential expansion and usage of interleukin-2. Immunology. (2014) 143(1):52–60. 10.1111/imm.12289 24673126 PMC4137955

[29] Zhou Q Qin S Zhang J Zhon L Pen Z Xing T. 1,25(OH)2D3 Induces regulatory T cell differentiation by influencing the VDR/PLC-γ1/TGF-β1/pathway. Mol Immunol. (2017) 91:156–64. 10.1016/j.molimm.2017.09.006 28926770

[30] Li B Zhang X Sun Z Xu B Wu J Liu H A novel strategy for the treatment of allergic rhinitis: regulating Treg/Th17 and Th1/Th2 balance in vivo by vitamin D. Comput Math Methods Med. (2022) 2022:9249627. 10.1155/2022/9249627 35959353 PMC9357782

[31] Marinho A Carvalho C Boleixa D Bettencourt A Leal B Guimarães J Vitamin D supplementation effects on FoxP3 expression in T cells and FoxP3+/IL-17A ratio and clinical course in systemic lupus erythematosus patients: a study in a Portuguese cohort. Immunol Res. (2017) 65(1):197–206. 10.1007/s12026-016-8829-3 27423437

[32] Thomas R Qiao S Yang X. Th17/Treg imbalance: implications in lung inflammatory diseases. Int J Mol Sci. (2023) 24(5):4865. 10.3390/ijms24054865 36902294 PMC10003150

[33] Bader DA Abed A Mohammad BA Aljaberi A Sundookah A Habash M The effect of weekly 50,000 IU vitamin D3 supplements on the serum levels of selected cytokines involved in cytokine storm: a randomized clinical trial in adults with vitamin D deficiency. Nutrients. (2023) 15(5):1188. 10.3390/nu15051188 36904187 PMC10005440

[34] Walawska-Hrycek A Galus W Hrycek E Kaczmarczyk A Krzystanek E. The impact of vitamin D low doses on its serum level and cytokine profile in multiple sclerosis patients. J Clin Med. (2021) 10(13):2781. 10.3390/jcm10132781 34202863 PMC8269072

[35] Farsani ZS Behmanesh M Sahraian MA. Interleukin-10 but not transforming growth factor-β1 gene expression is up-regulated by vitamin D treatment in multiple sclerosis patients. J Neurol Sci. (2015) 350(1–2):18–23. 10.1016/j.jns.2015.01.030 25680585

[36] Wei R Christakos S. Mechanisms underlying the regulation of innate and adaptive immunity by vitamin D. Nutrients. (2015) 7(10):8251–60. 10.3390/nu7105392 26404359 PMC4632412

[37] Athanassiou L Kostoglou-Athanassiou I Koutsilieris M Shoenfeld Y. Vitamin D and autoimmune rheumatic diseases. Biomolecules. (2023) 13(4):709. 10.3390/biom13040709 37189455 PMC10135889

[38] Iruretagoyena M Hirigoyen D Naves R Burgos PI. Immune response modulation by vitamin D: role in systemic lupus erythematosus. Front Immunol. (2015) 6:513. 10.3389/fimmu.2015.00513 26528285 PMC4600954

[39] Kim CS Han KD Jung JH Choi HS Bae EH Ma SK Incidence and risk factors for osteoporotic fractures in patients with systemic lupus erythematosus versus matched controls. Korean J Intern Med. (2021) 36(1):154–63. 10.3904/kjim.2018.378 31234614 PMC7820659

